# A Remorin Gene *SiREM6*, the Target Gene of SiARDP, from Foxtail Millet (*Setaria italica*) Promotes High Salt Tolerance in Transgenic *Arabidopsis*

**DOI:** 10.1371/journal.pone.0100772

**Published:** 2014-06-26

**Authors:** Jing Yue, Cong Li, Yuwei Liu, Jingjuan Yu

**Affiliations:** State Key Laboratory of Agrobiotechnology, College of Biological Sciences, China Agricultural University, Beijing, China; Institute of Genetics and Developmental Biology, Chinese Academy of Sciences, China

## Abstract

Remorin proteins (REMs) form a plant-specific protein family, with some REMs being responsive to abiotic stress. However, the precise functions of REMs in abiotic stress tolerance are not clear. In this study, we identified 11 remorin genes from foxtail millet (*Setaria italica*) and cloned a remorin gene, *SiREM6*, for further investigation. The transcript level of *SiREM6* was increased by high salt stress, low temperature stress and abscisic acid (ABA) treatment, but not by drought stress. The potential oligomerization of SiREM6 was examined by negative staining electron microscopy. The overexpression of *SiREM6* improved high salt stress tolerance in transgenic *Arabidopsis* at the germination and seedling stages as revealed by germination rate, survival rate, relative electrolyte leakage and proline content. The *SiREM6* promoter contains two dehydration responsive elements (DRE) and one ABA responsive element (ABRE). An ABA responsive DRE-binding transcription factor, *SiARDP*, and an ABRE-binding transcription factor, *SiAREB1*, were cloned from foxtail millet. SiARDP could physically bind to the DREs, but SiAREB1 could not. These results revealed that *SiREM6* is a target gene of SiARDP and plays a critical role in high salt stress tolerance.

## Introduction

Plant growth and development are constrained by environmental stress conditions. Salt stress is one of the major environmental stresses in agriculture worldwide and affects productivity and crop quality [1]. High salinity stress causes hyperosmotic stress, ion toxicity and nutrient deficiency, and can lead to molecular damage and even plant death. To respond and adapt to high salinity stress, plants have developed many strategies, such as selective ion uptake and exclusion, efficient detoxification by the antioxidant system [2], and the accumulation of osmotically protective matter [3]. Numerous salt tolerance- relevant genes are induced in response to salt stress [4].

The remorin protein family exists in all land plants, including angiosperms, gymnosperms, pteridophytes and bryophytes [5]. The first remorin was discovered in potato in 1989 and named pp34 for its 34 kD molecular mass position in protein gels [6]. The protein was renamed as remorin to indicate its ability to attach to the plasma membrane [7]. Recently, more remorin genes have been identified from different plants [8], [9], [10], [11], [12], [13]. Remorins contain a conserved C-terminal region and a variable N-terminal region. The coiled-coil structure exists in the C-terminal region of remorin and is considered the family’s signature. The variable N-terminal region of remorin suggests different structures and functions [14]. Based on the phylogenetic trees analysis and the different N-terminal domains, remorins are divided into six groups. While groups 1, 2 and 3 were not clearly separated by phylogeny, their domain features allowed them to be subdivided further [8]. In addition, many remorins could oligomerize *in vitro* [15].

Transcriptome and proteome analyses suggest that remorins play very important roles in plants in response to biotic and abiotic stresses [16], [12], [17], [18], [19], [20], [21]. A *Medicago truncatula* remorin protein, MtSYMREM1, induced during nodulation, interacts with symbiotic receptors, such as NFP, LYK3 and DMI2 that are important for the perception of bacterial signaling molecules. Oligomeric MtSYMREM1 attaches to the host plasma membrane surrounding the rhizobium, and controls the release of rhizobia into the host cytoplasm. Thus, *MtSYMREM1* has an important role during the plant-bacteria interaction [22]. Remorin gene *LjSYMREM1* was cloned from *Lotus japonicus*. The overexpression of *LjSYMREM1* increased root nodulation in transgenic plants. Functional analysis revealed that the C-terminal region of LjSYMREM1, especially the coiled-coil domain, was very important for protein interactions and remorin oligomerization. The RLK kinase interacted with the LjSYMREM1 protein *in vivo* and phosphorylated a residue in the N-terminal region *in vitro*. The molecular mechanisms of the LjSYMREM1 protein showed a new function and the importance of scaffold proteins during rhizobial infection [23]. *MiREM* from mulberry (*Morus indica*) was the first reported remorin gene involved in abiotic stress. Heterologous expression of MiREM in *Arabidopsis* enhanced drought and high salinity tolerance during the germination and seedling stages [5]. The study of abiotic stress-response functions for remorins in plants was novel.

The dehydration responsive element binding (DREB)-type transcription factors are a subfamily of the APETALA2 (AP2)/ethylene responsive factor (ERF) protein family, and play an important role in the responses to various stresses. Since the first DREB gene was cloned using the yeast one-hybrid screening system in *Arabidopsis* [24], [25], many DREB genes have been identified from rice, maize and barely [26], [27], [28]. Most DREB genes were responsive to abiotic stresses. The DREB proteins bind to the DRE core sequence in the promoter region of target genes and regulate their transcription. The overexpression of these DREB genes enhanced transgenic plant tolerance to abiotic stresses and accumulated osmoprotectants, such as proline and sugars [29]. Past studies indicate that DREB transcription factors regulated downstream gene expression through the abscisic acid (ABA)-independent signal pathway. However, increasing evidence shows that some DREB transcription factors are also responsive to ABA signals and are involved in the ABA signal pathway [30], [31].

Foxtail millet, an important crop in China, can grow in marginal soils and has a high tolerance of hostile environments [32]. It is important to identify new stress-relevant genes from foxtail millet. Although there is little data to confirm the function of the remorins in abiotic stresses, several expression analyses suggest that remorin genes are responsive to abiotic stresses and involved in signal transduction pathways [33], [34]. In the present study, we found 11 remorin genes, based on the C-terminal conserved domain of remorin proteins, in the foxtail millet transcriptome. We cloned them from foxtail millet cDNA and named them *SiREM1* to *SiREM11*. *SiREM6* was induced by high salinity, low temperature and ABA treatment. The overexpression of *SiREM6* in *Arabidopsis* enhanced the tolerance to high salt stress during seed germination and seedling development stages. *SiARDP*, an ABA responsive DREB transcription factor, can bind to DRE core elements in the promoter region of *SiREM6*. These results suggest that *SiREM6* is involved in salt tolerance under the control of the SiARDP transcription factor in the ABA-dependent signal pathway.

## Materials and Methods

### Plant materials and stress treatments

Foxtail millet (*Setaria italica*, cultivar Jigu 11) seeds were germinated on distilled water and grown in growth chambers (16 hour: 8 hour, light: dark cycle) at 28°C and 60% relative humidity. Two-week-old seedlings were transferred to 1/3 Hoagland solution, grown for 3 days, and then subjected to various stress treatments. Polyethylene glycol (PEG), NaCl and ABA treatments were conducted by transferring seedlings to 1/3 Hoagland solution containing 20% PEG6000, 150 mM NaCl and 100 µM ABA, respectively, and letting them grow for the indicated time. For the low temperature treatment, 17-day-old seedlings were transferred to a cold chamber and maintained at 4°C for the indicated time. For tissue expression analyses, the roots, stems and leaves were collected from 14-day-old untreated seedlings. The inflorescences were collected during the heading-stage from foxtail millet. These samples were frozen in liquid nitrogen, and then stored at −80°C.

### RNA extraction and RNA analysis

Total RNA from foxtail millet and *Arabidopsis* was extracted using TRIzol reagent (Invitrogen, Carlsbad, CA, USA). After digestion with RNase-free DNase I (Takara, Dalian, China), 2 µg of total RNA was converted into cDNA by M-MLV Reverse Transcriptase (Promega, Madison, WI, USA).

The reverse transcription polymerase chain reaction (RT-PCR) was performed using 2× Taq PCR StarMix with Loading Dye (GenStar, Beijing, China). PCR reactions were 95°C for 3 min, followed by 95°C for 30 sec, 60°C for 30 sec, 72°C for 30 sec for 25 cycles and 72°C for 5 min. Primers are listed in Table S1.

A quantitative real-time PCR (qRT-PCR) assay was performed using a LightCycler 480 II RT-PCR detection system (Roche, USA) with the UltraSYBR reagent mixture (CWBIO, Beijing, China). The PCR conditions were 95°C for 10 min, followed by 40 cycles of 95°C for 15 sec, 60°C for 1 min. The relative expression levels of mRNA were calculated using the ΔΔC_T_ method.

### Negative staining electron microscopy

The *SiREM6* and *StREM1* genes were cloned into the pET-28a vector containing a His tag. The recombinant vectors were independently transformed into *Escherichia coli* BL21 cells and then the cells were induced by 1 mM isopropyl-β-D-thiogalactoside (IPTG) for 4 h at 28°C. The fusion proteins were purified by nickel NTA (Qiagen, Germany).

For the negative-staining assay, recombinant remorins were dialyzed against 10 mM Tris (pH 7.5). The final protein concentrations were 80 µg/ml. Then, the recombinant remorins were adsorbed on formvar-coated copper grids for 10 min, stained with 2% uranyl acetate for 4 min, and air-dried. The samples were visualized at a magnification of 80000× using a Hitachi 7500 electron microscope (Japan). Photographs were taken using iTEM (OSIS, Germany).

### Generation of transgenic *Arabidopsis* plants

The full-length sequence of *SiREM6* was constructed in the modified binary vector pS1300 at the *Hin*dIII and *Xba*I sites controlled by the cauliflower mosaic virus (CaMV) 35S promoter. The constructed plasmid was introduced into *Agrobacterium tumefaciens* strain LBA4404 competent cells by the freeze-thaw method [35]. *Arabidopsis* plants were transformed by the vacuum infiltration method [36]. The transformed *Arabidopsis* seeds were screened on Murashige and Skoog (MS) medium containing 50 mg/L hygromycin. Three independent homozygous T3 seedling lines were chosen for subsequent experiments.

### Phenotypic analysis of transgenics

For the salt stress, approximately 80 seeds from the WT and each T3 generation of *Arabidopsis* lines were used for germination analysis. Surface sterilized seeds were sown on MS medium containing 0, 100, 150 and 175 mM NaCl for 7 days at 22°C. Then, the germination rate was scored and fresh/dry weights of the WT and transgenic *Arabidopsis* seedlings were measured.

For the early growth assay, 5-day-old WT and transgenic *Arabidopsis* seedlings were grown on MS medium and then transferred to MS medium containing 0, 150, 200 and 220 mM NaCl at 22°C. At 5 days, the survival rate was calculated.

For the growth assay, 7-day-old WT and transgenic *Arabidopsis* seedlings were grown on MS medium and then transferred to pots filled with soil and vermiculite (1∶1, v/v) for an additional 2 weeks. They were grown for 3 weeks using water containing 400 mM NaCl and then the survival rate was calculated.

Relative electrolyte leakage and proline content were measured as described by Zhao et al [37].

For the ABA treatment, approximately 6 seeds from the WT and each T3 generation of *Arabidopsis* lines were used for analysis. Surface sterilized seeds were sown on MS medium containing 0, 0.5, 0.75 and 1 µM ABA, and grown for 10 days at 22°C. Then the phenotype was observed.

For the dehydration stress, approximately 80 seeds from the WT and each T3 generation of *Arabidopsis* lines were used for analysis. Surface sterilized seeds were sown on MS medium containing 0 and 300 mM mannitol, and grown for 10 days at 22°C. Then the phenotype was observed.

The germination rate, survival rate, fresh/dry weights, relative electrolyte leakage and proline content data were subjected to Student’s t-test analysis using GraphPad Prism 5. All experiments were repeated three times.

### Electrophoretic mobility shift assay (EMSA)

Using their predicted sequences, *SiARDP* (SiPROV014314m) and *SiAREB1* (SiPROV013188m) were cloned from foxtail millet cDNA. The gene-specific primer pairs are listed in Table S1.

The *SiARDP* and *SiAREB1* genes were cloned into the pGEX-TEV vector containing a GST tag. The recombinant vectors were transformed into *E*. coli BL21 cells, and the cells were induced by 1 mM IPTG for 4 h at 28°C. Then, the fusion proteins were purified using Glutathione Sepharose 4B (GE, USA). Oligonucleotides and their reverse complementary oligonucleotides, which were labeled with biotin, were synthesized. Double-stranded DNA was obtained by heating oligonucleotides at 92°C for 30 sec, and annealing at 30°C. The gel-shift assay was performed following the manufacturer’s protocol for the LightShift Chemiluminescent EMSA Kit (Thermo, USA).

## Results

### The sequence characteristics of SiREM6

**Figure 1:**
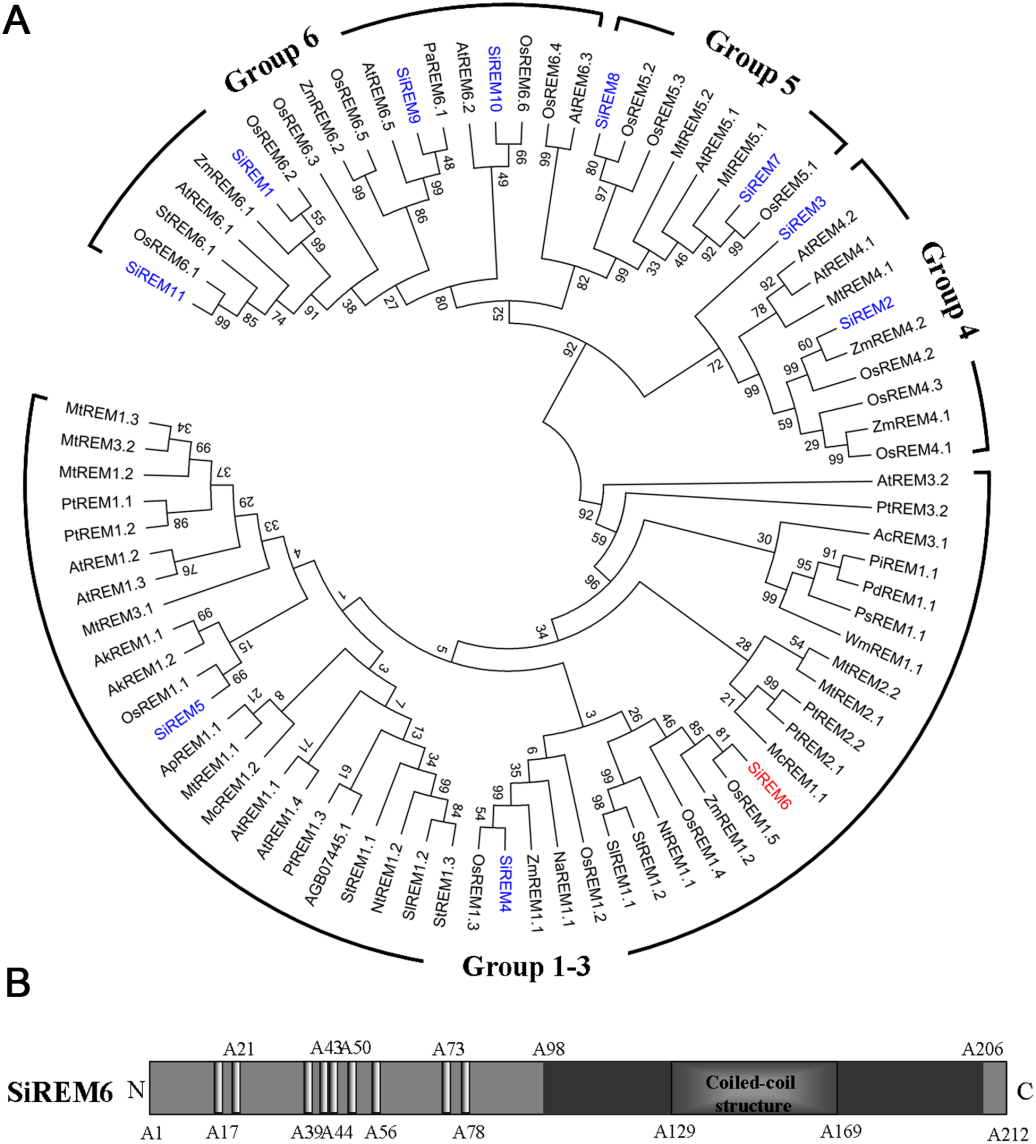
Phylogenetic tree of remorin proteins and predicted domains the SiREM6 protein. (A) Phylogenetic tree of remorin proteins from various plants. The multiple alignments were generated by MUSCLE and the phylogenetic tree was constructed by MEGA5.2.2 using a bootstrap test of phylogeny and the Neighbor Joining test with default parameters. The proteins belonged to four brackets: groups 1 to 3, group 4, group 5 and group 6. The nomenclature is based on Raffaele et al. (2007). The Genbank numbers of the remorin proteins used for the phylogenetic tree are shown in Table S2. (B) Predicted domains in the SiREM6 protein. The nine prolines in the N-terminal region are indicated as amino acid residue numbers. The black box indicates the C-terminal conserved region, and the coiled-coil structure is in this region between A129 and A169.

To isolate remorin genes from foxtail millet, the C-terminal conserved sequence of remorin was used as a query to search the foxtail millet transcriptome in the Phytozome and the *Setaria italica* databases. Based on the search results, 11 remorin genes were identified and named *SiREM1* to *SiREM11*. A phylogenetic analysis showed that these remorin could be divided into four subgroups. SiREM4, 5 and 6 belonged to groups 1 to 3, SiREM2 and 3 belonged to group 4, SiREM7 and 8 belonged to group 5, and SiREM1, 9, 10 and 11 belonged to group 6 (Fig. 1A). SiREM4, 5 and 6 were then classified into group 1 based on their N-terminal domain features [8]. It had been reported that the remorin proteins in group 1 might be involved in abiotic stresses [5]. The transcription levels of *SiREM4*, *5* and *6* in response to various stresses were analyzed by RT-PCR (data not shown). *SiREM6*, which responded to multiple treatments, was chosen for further analysis.

The open reading frame of *SiREM6* (SiPROV019639m) was cloned by PCR using sequence-specific primers as determined by the database analysis (Table S1). It contains 639 bp and encodes a protein of 212 amino acids with a predicted molecular mass of 23.1 kD. The isoelectric point is 5.40. SiREM6 contains the conserved C-terminal coiled-coil structure, a signature of remorin. Similar to OsREM1.5 and ZmREM1.2, which belong to group 1a, there are nine prolines in the N-terminal region of SiREM6 (Fig. 1B).

### Expression analysis of *SiREM6* under abiotic stresses

**Figure 2:**
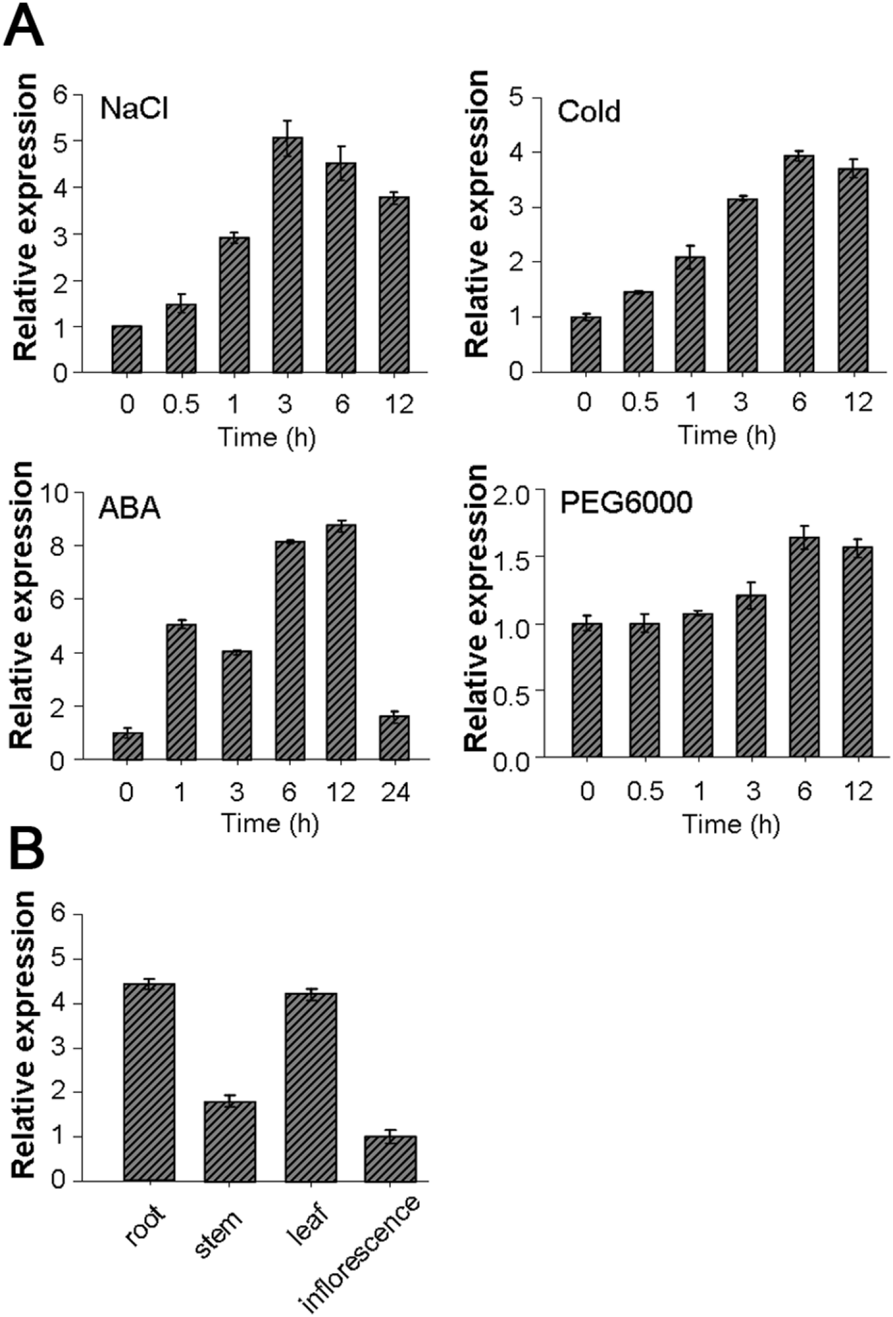
Expression patterns of the SiREM6 under different treatments and in different tissues of foxtail millet (*Setaria italica*). The 17-day-old foxtail millet seedlings subjected to NaCl (150 mM), cold (4°C), ABA (100 µM) and PEG (20% v/v) treatments for selected time periods. (A) Transcription levels of *SiREM6* in response to various stresses in foxtail millet seedlings as demonstrated by qRT-PCR. (B) Transcription levels of *SiREM6* in different tissues of foxtail millet seedlings as demonstrated by qRT-PCR. Foxtail millet *actin* (GenBank: AF288226) was amplified as a normalization control.

To analyze the expression patterns of *SiREM6* under different abiotic stresses and ABA treatment, qRT-PCR was conducted. The results showed that *SiREM6* expression was induced by ABA treatment, high salt and cold stresses, but not by drought stress (Fig. 2A). The transcription levels of *SiREM6* reached the highest level of 5.2-fold at 3 h, and maintained a similarly high level in the following 9 hours under 150 mM NaCl treatment. Under ABA treatment, the mRNA of *SiREM6* accumulated and reached 9.1-fold at 12 h, then decreased dramatically at 24 h. During cold treatment, *SiREM6* mRNA levels increased gradually and peaked at 6 h. To analyze the expression patterns in different tissues, total RNA isolated from different foxtail millet tissues were reverse transcribed as the templates for qRT-PCR. The results showed that *SiREM6* was expressed in root, stem, leaf and inflorescences (Fig. 2B).

### Oligomerization of SiREM6 *in vitro*

**Figure 3:**
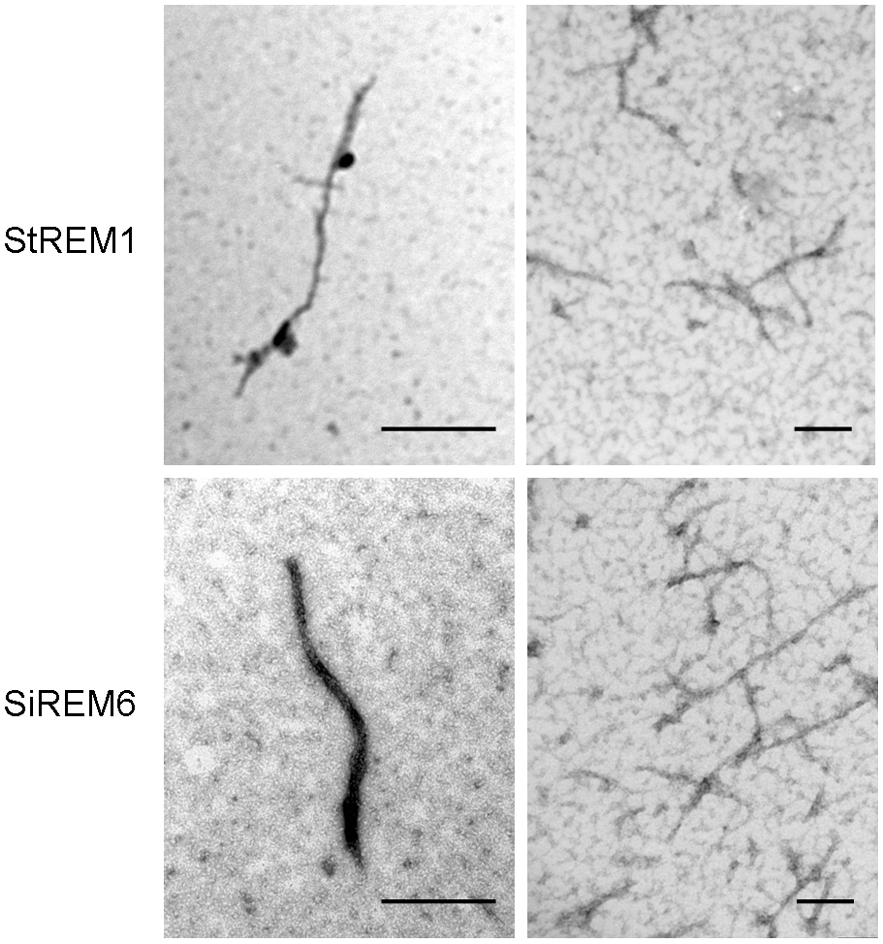
Electron microscopic images of remorin filaments. The protein samples were stained with uranyl acetate as described in the section “Materials and Methods”. StREM1: *Solanum tuberosum* remorin 1; SiREM6: *Setaria italica* remorin 6. Bar = 100 nm.

To examine the potential oligomerization of SiREM6, negative staining electron microscopy was performed. StREM1 from potato which could oligomerize *in vitro* was used as the positive control [15]. StREM1-His and SiREM6-His fusion proteins were purified and analyzed. Filamentous structures were clearly visible under electron microscopy (Fig. 3). These results suggest that, like other remorin proteins, SiREM6 could oligomerize.

### Functional analysis of *SiREM6* in transgenic *Arabidopsis*

**Figure 4:**
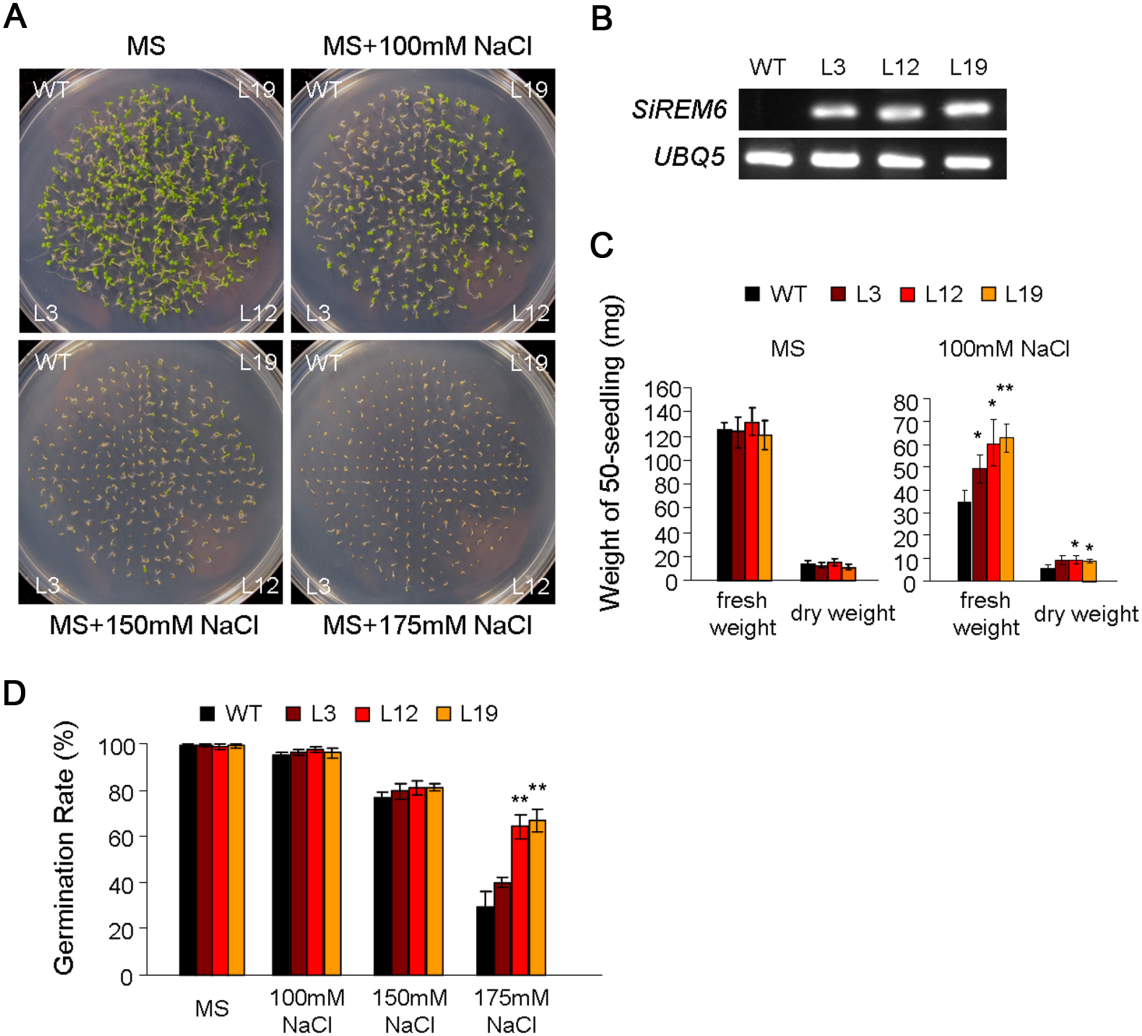
Overexpression of *SiREM6* improves salt stress tolerance in *Arabidopsis* during the germination stage. (A) NaCl stress tolerance of WT and transgenic plants. Seeds of WT and transgenic lines were germinated on medium containing 0 (control), 100, 150 and 175 mM NaCl for 7 days. (B) Transcription levels of SiREM6 in transgenic *Arabidopsis* and WT plants (negative control). *Arabidopsis UBQ5* (GenBank: AT3G62250) was amplified as a normalization control. (C) Fresh/dry weights of 7-day-old seedlings grown on medium containing 0 and 100 mM NaCl. (D) The germination rate of WT and transgenic lines on medium containing 0, 100, 150 and 175 mM NaCl for 7 days. Each data point had three replicates. For C and D, error bars indicate + SD. * and ** indicate statistically significant differences with *P*<0.05 and *P*<0.01 (Student’s *t*-test), respectively.

To analyze the function of *SiREM6* in stress tolerance, transgenic *Arabidopsis* plants expressing SiREM6 under the control of the CaMV 35S promoter were generated. A total of 30 independent transgenic *Arabidopsis* plants were obtained using a vacuum infiltration method. After RT-PCR analysis, three independent homozygous *SiREM6* overexpression T3 lines (L3, L12 and L19) were selected for further functional analyses (Fig. 4B).

To evaluate the influence of salt stress during the germination stage, seeds of WT and transgenic lines were sown on MS medium containing 0, 100, 150 and 175 mM NaCl. The WT and three transgenic lines did not show any difference on normal MS medium (Fig. 4A); however, the transgenic lines grew better than the WT on MS medium containing 100, 150 and 175 mM NaCl. The fresh/dry weight of seedlings indicated that the salt stress had a weaker influence on growth in the transgenic lines than in the WT plants (Fig. 4C). The germination rate was calculated for seeds on MS medium containing NaCl after 7 days. The germination rate showed no obvious differences between WT and transgenic lines on MS medium containing 100 and 150 mM NaCl. However, when the MS medium contained 175 mM NaCl, the germination rates of the transgenic lines were much higher than that of WT (Fig. 4D).

**Figure 5:**
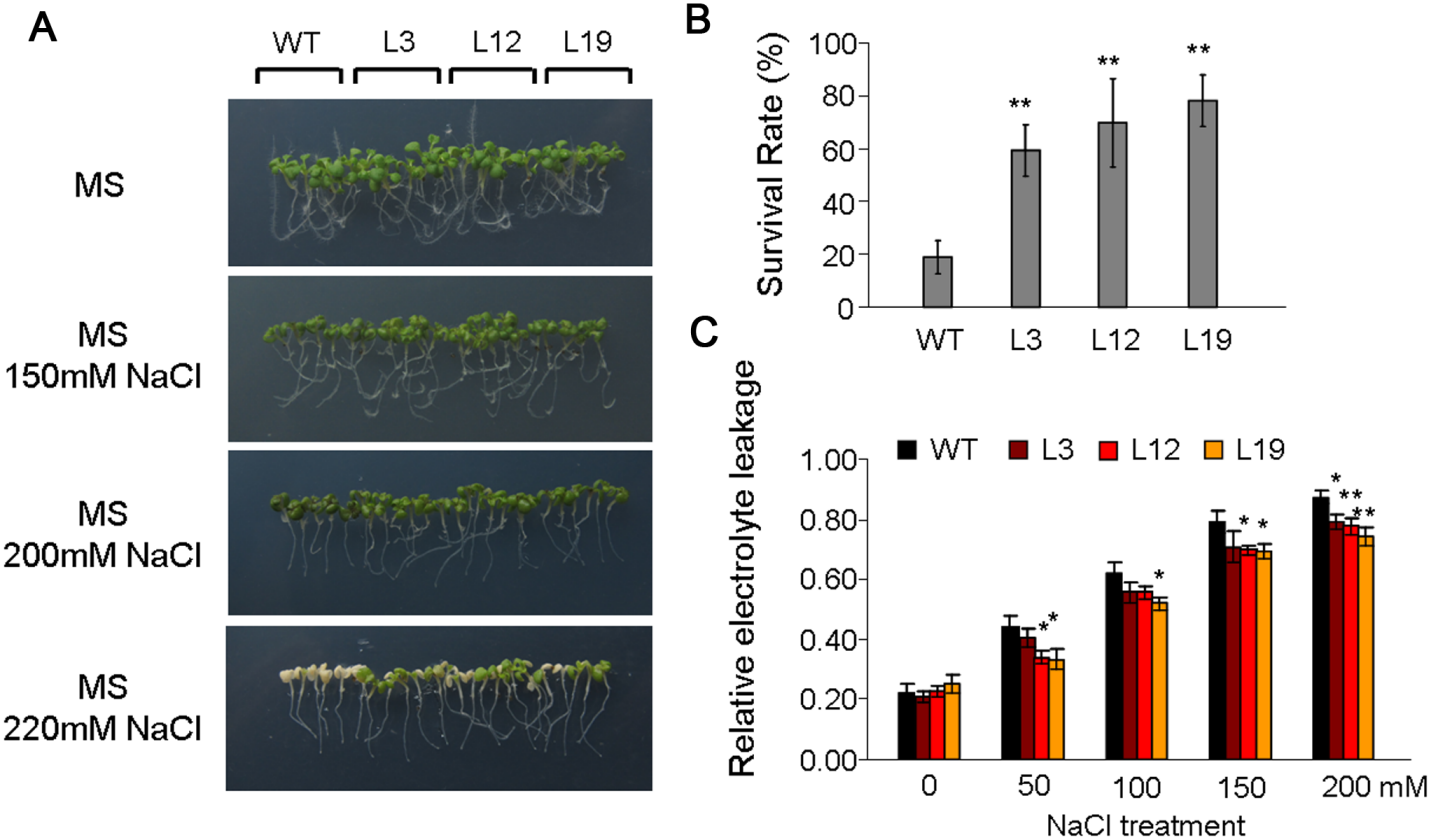
Overexpression of *SiREM6* improves salt stress tolerance in *Arabidopsis* during young seedling stage. (A) NaCl stress tolerance of WT and transgenic lines. Five-day-old seedlings were transferred to medium containing 0, 150, 200 and 220 mM NaCl for 5 days. (B) The survival rates of plants were analyzed after growing on medium containing 220 mM NaCl for 5 days. This experiment had three replicates, and each experiment comprised at least 30 plants. (C) The relative electrolyte leakage in WT and transgenic lines after exposure to different salt stress levels. Each data point had three replicates. For B and C, error bars indicate + SD. * and ** indicate statistically significant differences with *P*<0.05 and *P*<0.01 (Student’s *t*-test), respectively.

To evaluate the influence of salt stress during the seedling stage, the 5-day-old WT and transgenic seedlings grown under normal conditions were transferred to MS medium containing 0, 150, 200 and 220 mM NaCl, and maintained for 5 days. The growth rates of WT and transgenic lines were not obviously different on normal MS medium. When grown on the MS medium containing NaCl, the growth of the WT was affected more seriously than that of the transgenic lines, and more WT seedlings were bleached (Fig. 5A). The survival rates of the transgenic lines grown on medium containing 220 mM NaCl were significantly higher than those of the WT lines (Fig. 5B). The results of electrolyte leakage analysis showed that WT seedlings were more seriously damaged than transgenic seedlings under salt stress (Fig. 5C).

**Figure 6:**
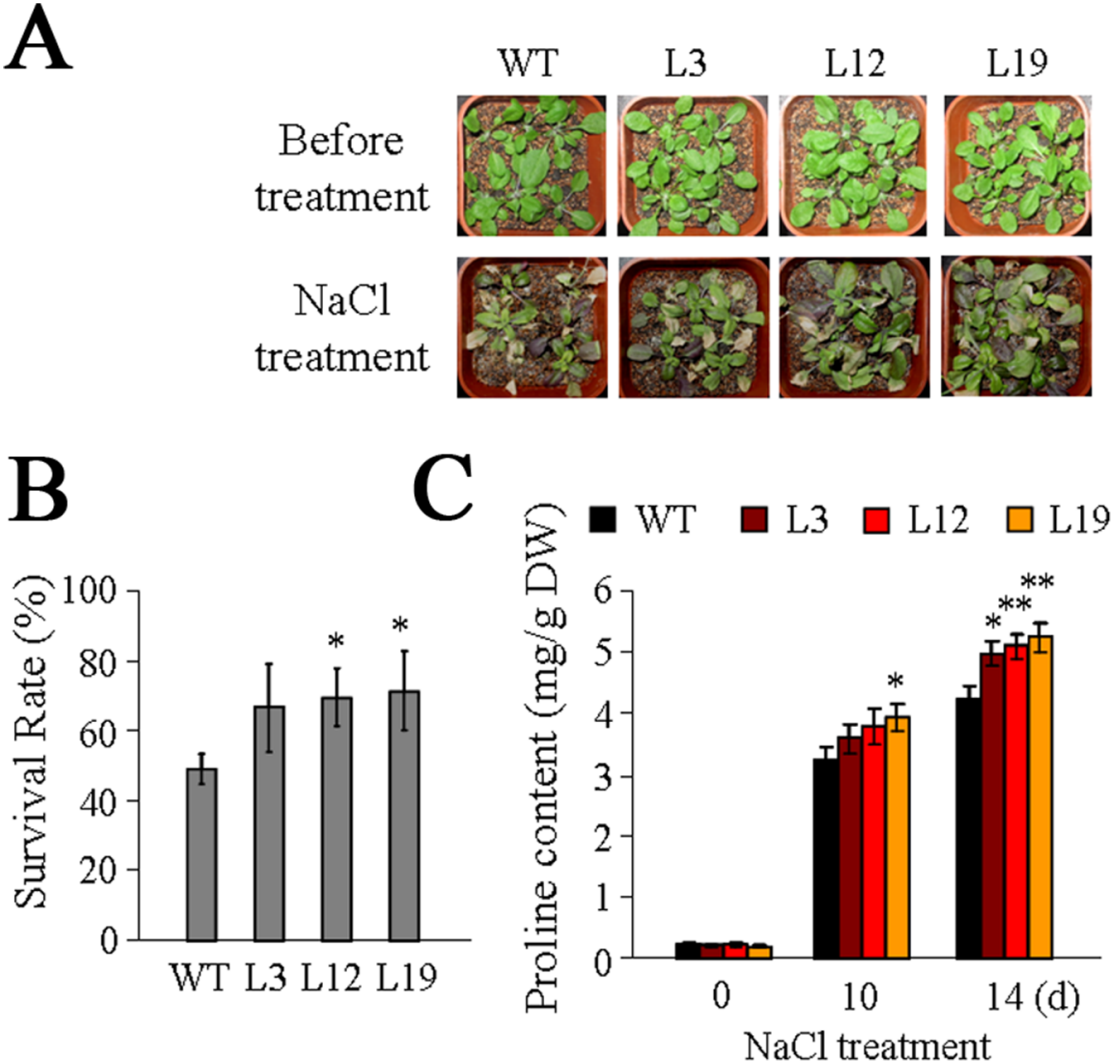
Overexpression of *SiREM6* improves salt stress tolerance in *Arabidopsis* during the seedling stage. (A) NaCl stress tolerance of WT and transgenic *Arabidopsis*. Two-week-old WT and transgenic *Arabidopsis* plants were treated with water containing 400 mM NaCl for three weeks. (B) The survival rates of plants were analyzed after treatment with water containing 400 mM NaCl for 3 weeks. This experiment had three replicates, and each experiment comprised at least 36 plants. (C) The proline content was analyzed in WT and transgenic plants after exposure to salt stress for 10 and 14 days. Each data point had three replicates. Error bars indicate + SD, and * and ** indicate statistically significant differences with *P*<0.05 and *P*<0.01 (Student’s *t*-test), respectively.

To further test the function of *SiREM6* during salt tolerance, 2-week-old WT and transgenic seedlings, grown under normal conditions, were treated with water containing 400 mM NaCl for three weeks. The WT seedlings were more significantly damaged than the transgenic seedlings (Fig. 6A). The survival rates of the transgenic lines were higher than 60%, while the survival rates of the WT were lower than 50% (Fig. 6B). The proline contents of WT and transgenic seedlings were measured after 14 days under the 400 mM NaCl stress treatment. The result showed that more proline had accumulated in the transgenic lines than in the WT (Fig. 6C).

As *SiREM6* expression was also induced by ABA treatment (Fig. 2A), the transgenic plants were tested for response to ABA treatment. The seeds of WT and trangenic lines were sown on MS medium containing 0, 0.5, 0.75 and 1 µM ABA, and grown for 10 days. The results were shown in Fig. S3. The WT and transgenic lines did not show any difference on normal MS medium, whereas the transgenic lines showed higher sensitivity to ABA treatment than the WT. The response of transgenic lines to drought was further detected even though the transcript level of *SiREM6* was weakly induced by drought stress. The seeds of WT and transgenic lines were sown on MS medium containing 0 and 300 mM mannitol, and grown for 10 days. As shown in Fig. S4, the WT and transgenic lines did not show any difference on MS containing 0 and 300 mM mannitol.

### Identification of *SiARDP* and *SiAREB1*

Because *SiREM6* was responsive to both salt stress and ABA treatment, we analyzed the promoter of *SiREM6* and found two DRE and one ABRE core elements (Fig. S1). The DREB transcription factors and AREB transcription factors bind the DRE and ABRE elements, respectively. To analyze the regulation of SiREM6, we cloned a DREB and an AREB transcription factor from foxtail millet, *SiARDP* (SiPROV014314m) and *SiAREB1* (SiPROV013188m), respectively. The SiARDP and SiAREB1 were located in the nucleus and had the ability of transcriptional activity in yeast (data not shown). The transcription levels of *SiARDP* and *SiAREB1* were also induced by salt stress and ABA treatment (Fig. S2).

### SiARDP binds to the promoter region of *SiREM6*

**Figure 7:**
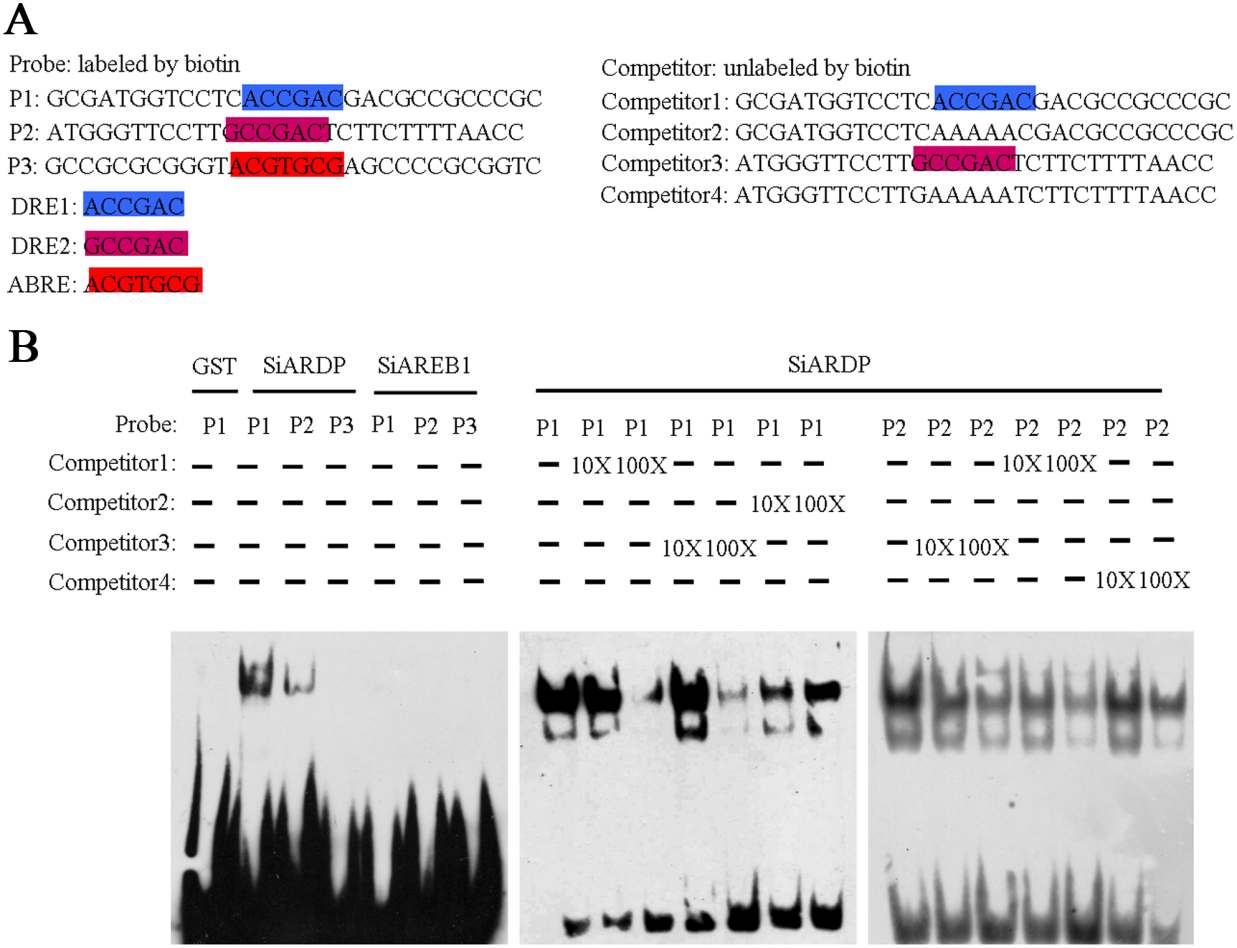
DNA binding abilities of dehydration responsive element (DRE)-binding transcription factor (TF), SiARDP, and abscisic acid responsive element (ABRE)-binding TF, SiAREB1, to the promoter of *SiREM6*. (A) The probes (P) were labeled with biotin, while the competitors were unlabeled. P1, 2 and 3 contained the dehydration responsive element 1 (DRE1), DRE2 and abscisic acid responsive element (ABRE), respectively. Competitors 1 and 2 contained DRE1 and mutant DRE1, in which ACCGAC was replaced with AAAAAC, respectively. Competitors 3 and 4 contained DRE2 and mutant DRE2, in which GCCGAC was replaced with GAAAAA, respectively. (B) SiARDP and SiAREB1 bind to elements. SiARDP binds to the elements in the presence of changing competitor concentrations.

To assess the consequences of SiARDP and SiAREB1 binding to the elements in the promoter region of *SiREM6*, an EMSA was performed. The sequences that defined DRE1, ACCGAC, and DRE2, GCCGAC, were used as probe 1 (P1) and probe 2 (P2), respectively. The sequence that defined ABRE, ACGTGCG, was used as probe 3 (P3) (Fig. 7A). SiARDP and SiAREB1 were expressed as glutathione S-transferase (GST) fusion proteins in *E*. coli. These fusion proteins were purified and used in the EMSA. The results showed that SiARDP could bind to P1 and P2, but not to P3. The binding affinity of SiARDP for P2 was weaker than for P1. With additional unlabeled probes (competitors 1 and 3), the SiARDP binding signals for P1 and P2 were reduced, but the addition of the mutant probes (competitors 2 and 4) did not obviously reduce the signals. SiAREB1 could not bind to any probes. These results showed that SiARDP specifically binds to two DRE elements in the promoter of *SiREM6*, but SiAREB1 does not.

## Discussion

In the past, most studies on the functions of remorins focused on plant-microbe interactions and biotic stresses. However, the precise functions of remorins are not certain. Additionally, compared with their functions during biotic stress, less data has been reported on the functions of remorins during abiotic stress. Foxtail millet is an important crop in China. It is nutritionally rich and adapts well to stress [38]. However, there is less research on foxtail millet than on other crops, such as rice, maize and wheat. In the present study, 11 remorin genes were identified and cloned from foxtail millet, and the function of *SiREM6* during abiotic stress was analyzed.

The coiled-coil structure in the conserved C-terminal of proteins is a typical remorin signature. Proteins containing the coiled-coil structure usually interact with other coiled-coil proteins and can be oligomerized [39]. The SiREM6 protein contains the signature coiled-coil domain in the C-terminal and could be oligomerized *in vitro* (Fig. 3B). The signature domain features and similarities between remorin proteins combined with a phylogenetic analysis indicated that the remorin family is subdivided into six separate groups, and that SiREM6 is classified as belonging to group 1. The remorins in group 1 are subdivided into groups 1a and 1b according to the number of prolines in their N-terminal region [8]. SiREM6 contains nine prolines in its N-terminal region, thus SiREM6 belongs to the 1a subgroup. Many remorins in group 1a respond to abiotic stress and ABA treatment, and are involved in abiotic stress [5].

The expression levels of certain stress responsive genes may be associated with stress tolerance [40]. The transcription levels of *SiREM6* increased under NaCl, cold stress and ABA treatment (Fig. 2A). That this response to salt stress occurred rapidly, and was maintained at a high level, strongly implies that the function of *SiREM6* may involve the adaptation to salt stress. The germination rate and live weight are typical physiological parameters for evaluating plant resistance during the germination stage. Relative electrolyte leakage is a relevant index for measuring the cell damage of plants under stresses, and the accumulation of free proline plays a protective role in plants under various stresses. *SiREM6* overexpressing transgenic lines have a higher germination rate and live weight (Fig. 4C and D), and, after high salinity treatments, these transgenic lines had low relative electrolyte leakage and high levels of proline content (Figs. 5C and 6C). These results indicated that *SiREM6* expression in *Arabidopsis* reduced cellular injuries and made the transgenic lines more adaptable to salt stress during the germination and seedling stages. The NaCl stress includes ionic (Na^+^-specific) and osmotic stresses. The SiREM6 expression was weakly induced by drought, and SiREM6 transgenic lines could not improve the drought tolerance. This implied that the function of remorin proteins may be in resist ion stress.

The plant hormone ABA plays an important role in plants under abiotic stresses [41], [42], [43], and many remorin genes were induced by ABA treatment [33]. The promoter region of *SiREM6* contained two DRE elements and one ABRE element (Fig. S1). The results of qRT-PCR showed that *SiREM6* was also rapidly induced by ABA treatment (Fig. 2A) and the SiREM6 transgenic lines showed higher sensitive to ABA (Fig. S3). The DREB and AREB transcription factors are important regulatory factors in plants and regulate the expression of target genes during abiotic stress. We cloned the ABA response DRE-binding transcription factor, *SiARDP* and ABA response element (ARE)-binding transcription factor, *SiAREB1*, from foxtail millet. The transcription levels of *SiARDP* and *SiAREB1* were induced by salt stress and ABA treatments (Fig. S2). SiARDP binds to two DRE elements in the promoter region of *SiREM6*, while SiAREB1 did not bind to the ABRE element. These results suggest that SiARDP, but not SiAREB1, regulate the *SiREM6* gene in foxtail millet. SiARDP had a higher affinity for the DRE1 element in P1 than for the DRE2 element in P2. The difference in the core sequences, which occurs at the first base pair (A/G), may explain the difference in the binding affinity. These results indicate that *SiREM6* may be regulated by SiARDP in foxtail millet when under salt stress, and may be involved in the ABA-dependent pathway.

In addition, phosphorylation is a very important process in many abiotic stress signaling pathways. Remorin proteins have been reported to be phosphorylated *in vivo* [44], [45], [46]. The conserved C-terminal of remorin proteins could provide a stable structure for phosphorylation. Phosphorylation may change the conformation of remorin proteins, and then the changed remorins could interact with other proteins [23] to response the stresses. Further phosphorylation analysis of the SiREM6 will be helpful to deeply understand the molecular mechanism of SiREM6 in response to the stress.

Remorin genes exist extensively in plants, and have different functions in plants. We focused on the function of *SiREM6* in salt stress tolerance in foxtail millet. The expression of *SiREM6* is regulated by transcription factors under salt stress, including SiARDP. Overexpression of *SiREM6* could enhance salt stress tolerance in transgenic *Arabidopsis* plants. These processes rely on the accumulation of protective materials, such as proline, thereby reducing the damage to plant cells. Although the precise mechanism involving *SiREM6* during salt stress is not clear, our results demonstrated that *SiREM6* is involved in salt stress tolerance in plants.

## Supporting Information

Figure S1 **The** ***cis*** **-elements, dehydration responsive element (DRE) and abscisic acid responsive element (ABRE), identified in the** ***SiREM6*** **’s promoter.** The DRE1 (blue bar), DRE2 (purple bar), AREB (red bar) and TATA box (yellow bar) are shown.
(DOC)

Figure S2 **Expression pattern assay of dehydration responsive element (DRE)-binding transcription factor (TF),** ***SiARDP*** **, and an abscisic acid responsive element (ABRE)-binding TF,** ***SiAREB1*** **, under salt stress and abscisic acid (ABA) treatment in foxtail millet (** ***Setaria italica*** **).** The 17-day-old foxtail millet seedlings were treated with NaCl (150 mM) and ABA (100 µM) for selected time periods. (A) Transcription levels of *SiARDP* in response to NaCl stress and ABA treatment as demonstrated by qRT-PCR. (B) Transcription levels of *SiAREB1* in response to NaCl stress and ABA treatment as demonstrated by qRT-PCR.
(DOC)

Figure S3 **Overexpression of** ***SiREM6*** **enhances sensitivity to ABA treatment.** Seeds of WT and transgenic lines were sown on MS medium containing 0 (control), 0.5, 0.75 and 1 µM ABA, and grown under normal condition for 10 days.
(DOC)

Figure S4 **No difference between** ***SiREM6*** **transgenic lines and wild type under dehydration stress.** Seeds of WT and transgenic lines were sown on MS medium containing 0 (control) and 300 mM mannitol, and grown under normal condition for 10 days.
(DOC)

Table S1 Gene specific primers used in this study.
(DOC)

Table S2 The GenBank accession numbers of proteins used to develop the remorin phylogenetic tree.
(DOC)
