# Supplementary material for: A Remorin Gene SiREM6, the Target Gene of SiARDP, from Foxtail Millet (Setaria italica) Promotes High Salt Tolerance in Transgenic Arabidopsis
Source: PLoS One. 2014 Jun 26;9(6):e100772. doi: 10.1371/journal.pone.0100772 (PMC4072699; doi:10.1371/journal.pone.0100772)
Supplement: Figure S1 — The cis -elements, dehydration responsive element (DRE) and abscisic acid responsive element (ABRE), identified in the SiREM6 ’s promoter. The DRE1 (blue bar), DRE2 (purple bar), AREB (red bar) and TATA box (yellow bar) are shown. (DOC) [file pone.0100772.s001.doc]

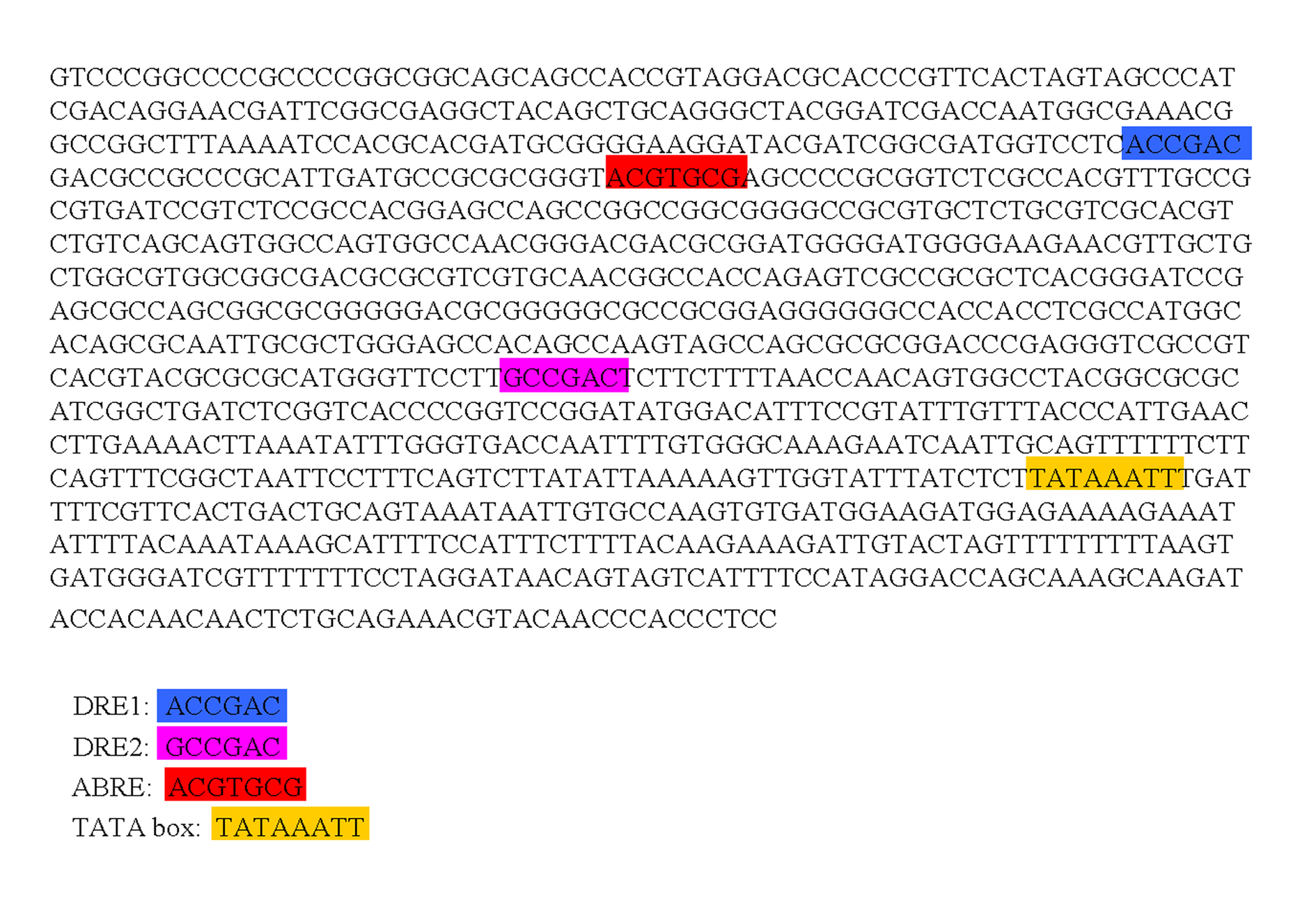


**Figure S1 DRE and ABRE *cis*-elements indentified in the *SiREM6* promoter.**

The DRE1 (blue bar), DRE2 (purple bar), ABRE (red bar) and TATA box (yellow bar) are shown.
