# Supplementary material for: A Remorin Gene SiREM6, the Target Gene of SiARDP, from Foxtail Millet (Setaria italica) Promotes High Salt Tolerance in Transgenic Arabidopsis
Source: PLoS One. 2014 Jun 26;9(6):e100772. doi: 10.1371/journal.pone.0100772 (PMC4072699; doi:10.1371/journal.pone.0100772)
Supplement: Figure S2 — Expression pattern assay of dehydration responsive element (DRE)-binding transcription factor (TF), SiARDP , and an abscisic acid responsive element (ABRE)-binding TF, SiAREB1 , under salt stress and abscisic acid (ABA) treatment in foxtail millet ( Setaria italica ). The 17-day-old foxtail millet seedlings were treated with NaCl (150 mM) and ABA (100 µM) for selected time periods. (A) Transcription levels of SiARDP in response to NaCl stress and ABA treatment as demonstrated by qRT-PCR. (B) Transcription levels of SiAREB1 in response to NaCl stress and ABA treatment as demonstrated by qRT-PCR. (DOC) [file pone.0100772.s002.doc]

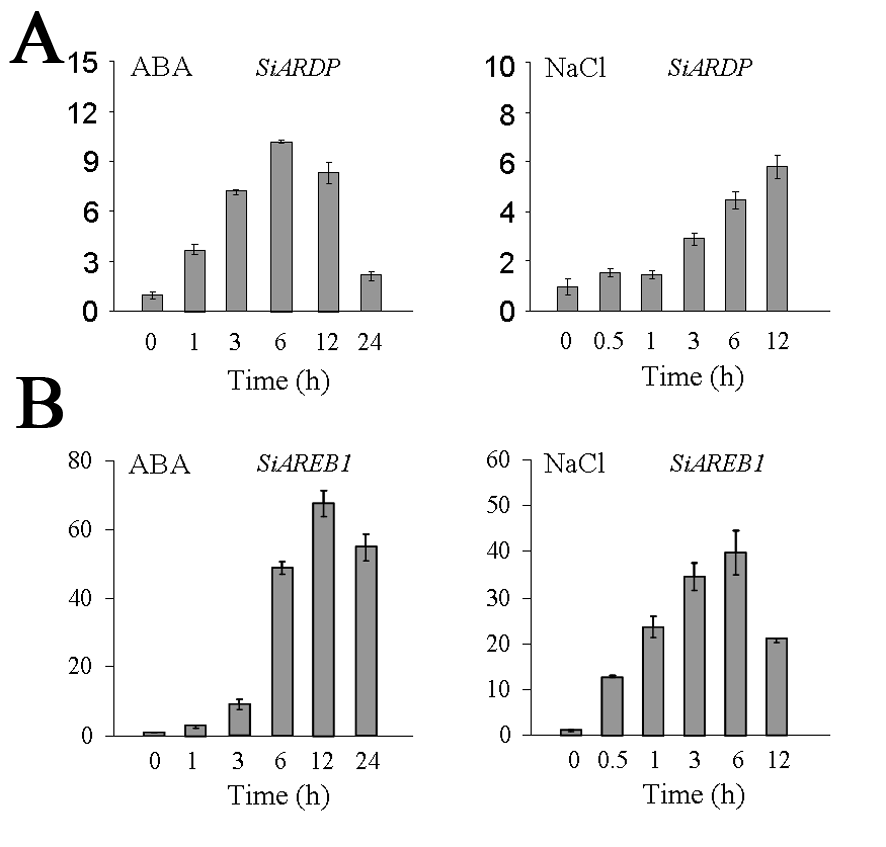


**Figure S2 Expression pattern assay of *SiARDP* and *SiAREB1* under salt stress and ABA treatment.**

The 17-day-old foxtail millet seedlings were treated under NaCl (150 mM) and ABA (100μM) at selected time points. (A) Transcription levels of *SiARDP* in response to NaCl stress and ABA treatment in foxtail millet seedlings as demonstrated by qRT-PCR. (B) Transcription levels of *SiAREB1* in response to NaCl stress and ABA treatment in foxtail millet seedlings as demonstrated by qRT-PCR.
