# Supplementary material for: A Remorin Gene SiREM6, the Target Gene of SiARDP, from Foxtail Millet (Setaria italica) Promotes High Salt Tolerance in Transgenic Arabidopsis
Source: PLoS One. 2014 Jun 26;9(6):e100772. doi: 10.1371/journal.pone.0100772 (PMC4072699; doi:10.1371/journal.pone.0100772)
Supplement: Figure S4 — No difference between SiREM6 transgenic lines and wild type under dehydration stress. Seeds of WT and transgenic lines were sown on MS medium containing 0 (control) and 300 mM mannitol, and grown under normal condition for 10 days. (DOC) [file pone.0100772.s004.doc]

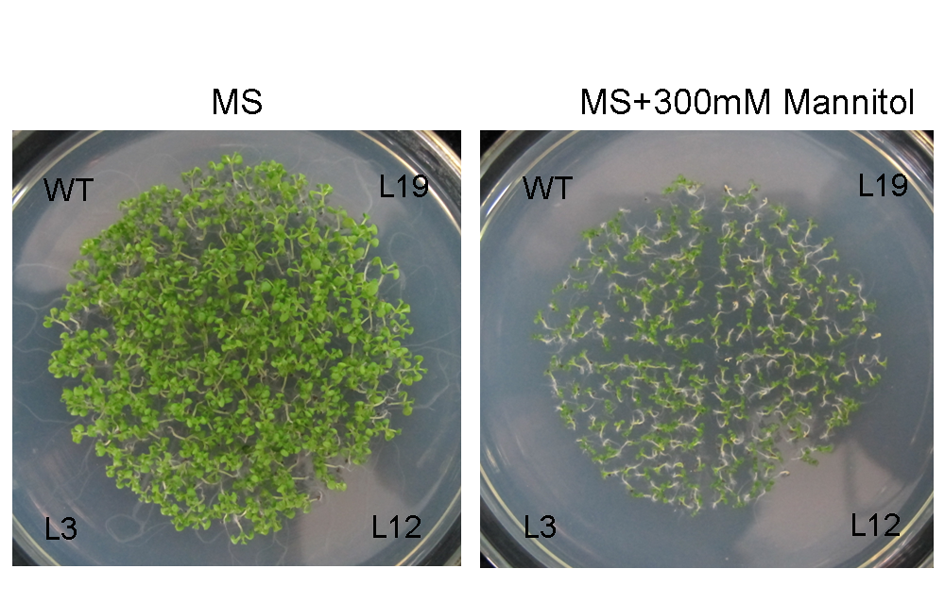


**Figure S4. No difference between *SiREM6* transgenic lines and wild type under dehydration stress.**

Seeds of WT and transgenic lines were sown on MS medium containing 0 (control) and 300 mM mannitol, and grown under normal condition for 10 days.
