# Supplementary material for: A Remorin Gene SiREM6, the Target Gene of SiARDP, from Foxtail Millet (Setaria italica) Promotes High Salt Tolerance in Transgenic Arabidopsis
Source: PLoS One. 2014 Jun 26;9(6):e100772. doi: 10.1371/journal.pone.0100772 (PMC4072699; doi:10.1371/journal.pone.0100772)
Supplement: Table S1 — Gene specific primers used in this study. (DOC) [file pone.0100772.s005.doc]

**Table S1 Gene specific primers used in this study**

| Table. S1 Primers sequences used in this study. | | |
| --- | --- | --- |
| Gene name | Forward primer | Reverse primer |
| *SiREM6* (qRT-PCR) | 5'-GCCGAGAAGATGAAGAACAAA-3 | 5'-AGGCTCCAAAGCACCCGATGA-3 |
| *SiREM6* (full-long ) | 5'-GGGGTACCATGGCTGAGGAGGCG-3 | 5'-CGGGATCCTTAGGCTCCAAAGCA-3 |
| *SiARDP* (qRT-PCR) | 5'-TGCGAGTCAACGACAACAT-3' | 5'-CAAAGCCAGCAACGAACAT-3' |
| *SiARDP* (full-long ) | 5'-ATGACGGTGGATCAGAAGCA-3' | 5'-CAGGCCCTCGAAGAAACCTG-3' |
| *SiAREB1* (qRT-PCR) | 5'-GGAGATCACGCTGGAGGAGT-3' | 5'-AGGCATCAATGGAGCAAACA-3' |
| *SiAREB1* (full-long ) | 5'-ATGGATTTCCCGGGCGGGAGCG-3' | 5'-TCAGCCAAGTTTGTTACTAGTA-3' |
| *Siactin* (qRT-PCR) | 5'-GTGCTTTCCCTCTACGCCAGTG-3' | 5'-ACCGCTGAGCACAATGTTACCA-3' |
| *UBQ5* (qRT-PCR) | 5'-CTCCTTCTTTCTGGTAAACGT-3' | 5'-GGTGCTAAGAAGAGGAAGAAT-3' |
