# Supplementary material for: A Remorin Gene SiREM6, the Target Gene of SiARDP, from Foxtail Millet (Setaria italica) Promotes High Salt Tolerance in Transgenic Arabidopsis
Source: PLoS One. 2014 Jun 26;9(6):e100772. doi: 10.1371/journal.pone.0100772 (PMC4072699; doi:10.1371/journal.pone.0100772)
Supplement: Table S2 — The GenBank accession numbers of proteins used to develop the remorin phylogenetic tree. (DOC) [file pone.0100772.s006.doc]

**Table S2 The GenBank accession numbers of proteins used to develop the remorin phylogenetic tree**

| Table S2 The GenBank accession numbers of proteins used for phylogenetic tree. | | |
| --- | --- | --- |
| Proposed nomenclature | Species | Gene / EST number |
| AcREM3.1 | Ferns | TA1841_13818 |
| AkREM1.1 | Amborellaceaes | 200125 |
| AkREM1.2 | Amborellaceaes | CO995618 |
| ApREM1.1 | Liliales | CF445491 |
| AtREM1.1 | Brassicales | AT3G48940 |
| AtREM1.2 | Brassicales | AT3G61260 |
| AtREM1.3 | Brassicales | AT2G45820 |
| AtREM1.4 | Brassicales | AT5G23750 |
| AtREM3.2 | Brassicales | AT4G00670 |
| AtREM4.1 | Brassicales | AT3G57540 |
| AtREM4.2 | Brassicales | AT2G41870 |
| AtREM5.1 | Brassicales | AT1G45207 |
| AtREM6.1 | Brassicales | AT2G02170 |
| AtREM6.2 | Brassicales | AT1G30320 |
| AtREM6.3 | Brassicales | AT1G53860 |
| AtREM6.5 | Brassicales | AT1G67590 |
| McREM1.1 | Caryophyllales | 402614 |
| McREM1.2 | Caryophyllales | BF480549 |
| MtREM1.1 | Fabales | MtC60319 |
| MtREM1.2 | Fabales | MtC00278 |
| MtREM1.3 | Fabales | MtD17655 |
| MtREM2.1 | Fabales | MtD05125 |
| MtREM2.2 | Fabales | MtC10811 |
| MtREM3.1 | Fabales | ABE84731 |
| MtREM3.2 | Fabales | MtD26156 |
| MtREM4.1 | Fabales | MtC93157 |
| MtREM5.1 | Fabales | MtD03479 |
| MtREM5.2 | Fabales | MtD17032 |
| NaREM1.1 | Nymphaeaceaes | CD473731 |
| NtREM1.1 | Solanales | KT7C.109M13 |
| NtREM1.2 | Solanales | KT7C.104L22 |
| OsREM1.1 | Poales | Os02g57840 |
| OsREM1.2 | Poales | Os10g36000 |
| OsREM1.3 | Poales | Os03g02040 |
| OsREM1.4 | Poales | Os02g42880 |
| OsREM1.5 | Poales | Os04g45070 |
| OsREM4.1 | Poales | Os07g38170 |
| OsREM4.2 | Poales | Os03g59360 |
| OsREM4.3 | Poales | Os07g10780 |
| OsREM5.1 | Poales | Os02g52810 |
| OsREM5.2 | Poales | Os08g36760 |
| OsREM5.3 | Poales | Os09g28300 |
| OsREM6.1 | Poales | Os02g02500 |
| OsREM6.2 | Poales | Os03g02840 |
| OsREM6.3 | Poales | Os12g41940 |
| OsREM6.4 | Poales | Os11g40210 |
| OsREM6.5 | Poales | Os02g39000 |
| OsREM6.6 | Poales | Os04g52920 |
| PaREM6.1 | Laurales | 209963 |
| PdREM1.1 | Pinales | TA9116_3352 |
| PiREM1.1 | Pinales | BX254033 |
| PsREM1.1 | Pinales | TA11262_3332 |
| PtREM1.1 | Malpighiales | fgenesh4_pm.C_LG_XIV0110 |
| PtREM1.2 | Malpighiales | grail3.0039003901 |
| PtREM1.3 | Malpighiales | fgenesh4_pm.C_LG_XV0452 |
| PtREM2.1 | Malpighiales | fgenesh4_pg.C_LG_I0941 |
| PtREM2.2 | Malpighiales | eugene3.00030969 |
| PtREM3.1 | Malpighiales | eugene3.00150257 |
| SlREM1.1 | Solanales | SlREM2 |
| SlREM1.2 | Solanales | SlREM1 |
| StREM1.1 | Solanales | BI176676 |
| StREM1.2 | Solanales | DN921711 |
| StREM1.3 | Solanales | DN921712 |
| StREM6.1 | Solanales | 05Mar7.CPGP.8199.C2 |
| WmREM1.1 | Gnetales | 351201 |
| ZmREM1.1 | Poales | TA104935_4577 |
| ZmREM1.2 | Poales | CF004505 |
| ZmREM4.1 | Poales | TA114130_4577 |
| ZmREM4.2 | Poales | TA119747_4577 |
| ZmREM6.1 | Poales | TA117003_4577 |
| ZmREM6.2 | Poales | TA135644_4577 |
| AGB07445.1 | Rosales | AGB07445.1 |
| SiREM1 | Poales | SiPROV019369m |
| SiREM2 | Poales | SiPROV016496m |
| SiREM3 | Poales | SiPROV009546m |
| SiREM4 | Poales | SiPROV020674m |
| SiREM5 | Poales | SiPROV018885m |
| SiREM6 | Poales | SiPROV019639m |
| SiREM7 | Poales | SiPROV034318m |
| SiREM8 | Poales | SiPROV010077m |
| SiREM9 | Poales | SiPROV013923m |
| SiREM10 | Poales | SiPROV006635m |
| SiREM11 | Poales | SiPROV006913m |
